# 2-Chloro-*N*-(4-hy­droxy­phen­yl)acetamide

**DOI:** 10.1107/S2414314624010150

**Published:** 2024-10-24

**Authors:** Abderrazzak El Moutaouakil Ala Allah, Benson M. Kariuki, Issam Ameziane El Hassani, Abdulsalam Alsubari, Walid Guerrab, Musa A. Said, Youssef Ramli

**Affiliations:** a Laboratory of Medicinal Chemistry Drug Sciences Research Center Faculty of Medicine and Pharmacy Mohammed V University in Rabat Morocco; b School of Chemistry, Cardiff University, Main Building, Park Place, Cardiff, CF10 3AT, United Kingdom; c Laboratory of Medicinal Chemistry, Faculty of Clinical Pharmacy, 21 September University, Yemen; d Department of Chemistry Faculty of Science Islamic University of Madinah, Madinah 42351 Saudi Arabia

**Keywords:** crystal structure, acetamide, hydrogen-bonding

## Abstract

The title compound, C_8_H_8_ClNO_2_, is significantly distorted from planarity, with a twist angle between the planes through the hy­droxy­benzene and acetamide groups being 23.5 (2)°. This conformation is supported by intra­molecular C—H⋯O and N—H⋯Cl contacts. In the crystal, N—H⋯O hydrogen-bonding contacts between acetamide groups and O—H⋯O contacts between hydroxyl groups form tapes propagating parallel to [103].

## Abstract 2

The title compound is significantly distorted from planarity, with a twist angle between the planes through the hy­droxy­benzene and acetamide groups being 23.5 (2)°. This conformation is supported by intra­molecular C—H⋯O and N—H⋯Cl contacts. In the crystal, N—H⋯O hydrogen-bonding contacts between acetamide groups and O—H⋯O contacts between hydroxyl groups form tapes propagating parallel to [103].

## Graphical abstract

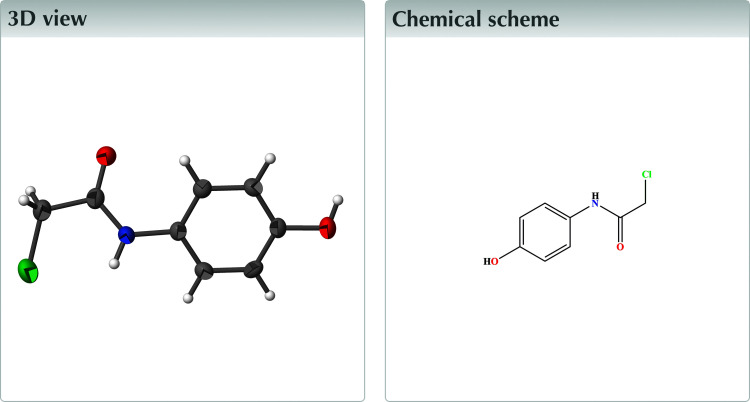

## Structure description

*N*-aryl­acetamides are inter­mediates for the synthesis of medicinal, agrochemical and pharmaceutical compounds (Missioui *et al.*, 2021 ▸). As part of our ongoing studies of these systems (Missioui *et al.*, 2022 ▸), we now describe the synthesis and structure of the title compound, C_8_H_8_ClNO_2_.

**Figure 1:**
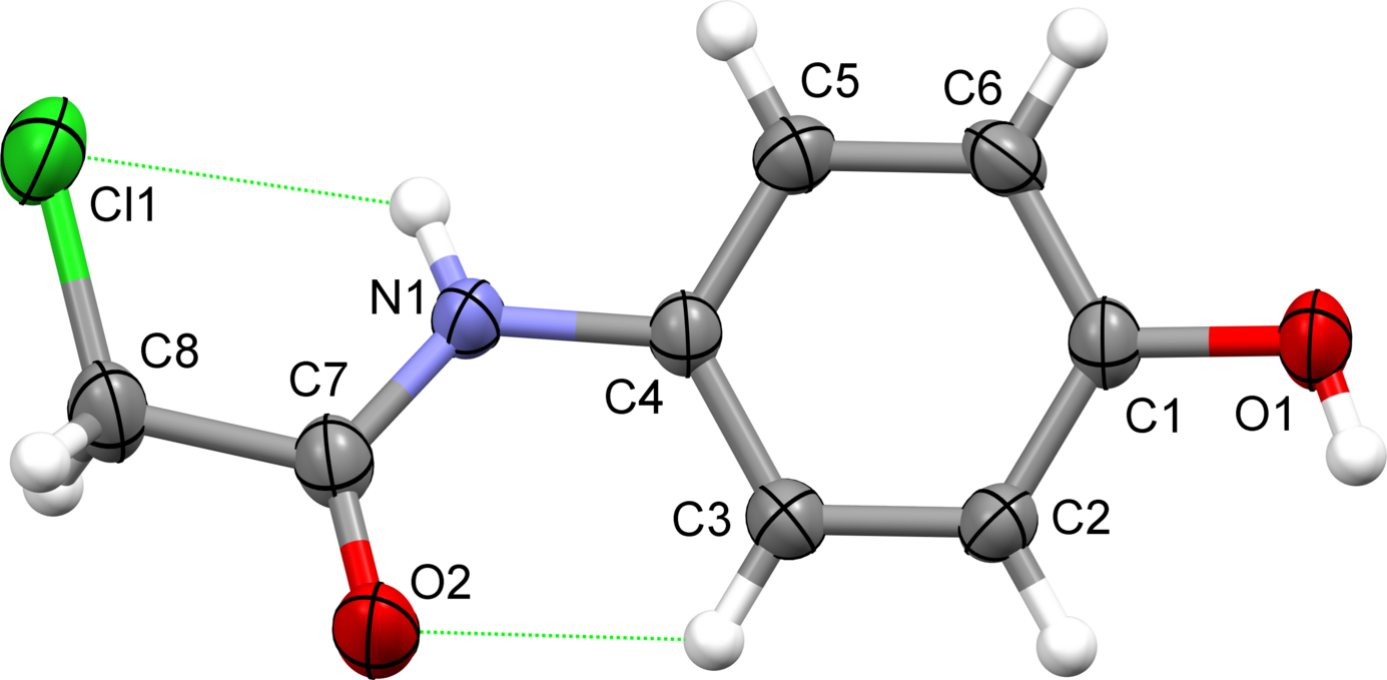
The mol­ecule of 2-chloro-*N*-(4-hy­droxy­phen­yl)acetamide showing the atom-numbering scheme and displacement parameters at the 50% probability level. The intra­molecular C—H⋯O and N—H⋯Cl contacts are shown as green dotted lines.

The mol­ecule, Fig. 1 ▸, is almost planar as indicated by a twist angle between the planes through the hy­droxy­benzene (C1–C6, O1) and acetamide (C7, C8, N1, O2) groups being 23.5 (2)°; the acetamide group has an *anti* conformation. The chloro substituent deviates only slightly from the plane of the acetamide group as indicated by the N1—C7—C8—Cl1 torsion angle of 15.4 (4)°.

Two types of close intra­molecular contacts occur within the mol­ecule. The first contact is of the type C—H⋯O with a C3—H3⋯O2 angle of 116° and a C3⋯O2 distance of 2.873 (4) Å, Table 1 ▸. Similar contacts are observed in related structures including 2-chloro-*N*-(4-fluoro­phen­yl)acetamide (Kang *et al.*, 2008 ▸), 2-chloro-*N*-phenyl­acetamide (Gowda *et al.*, 2008 ▸) and 2-chloro-*N*-(4-chloro­phen­yl)acetamide (Gowda *et al.*, 2007 ▸). The second contact is of the type N—H⋯Cl and has a N1—H1⋯Cl1 angle of 115° and a N1⋯Cl1 distance of 2.999 (2) Å.

**Figure 2:**
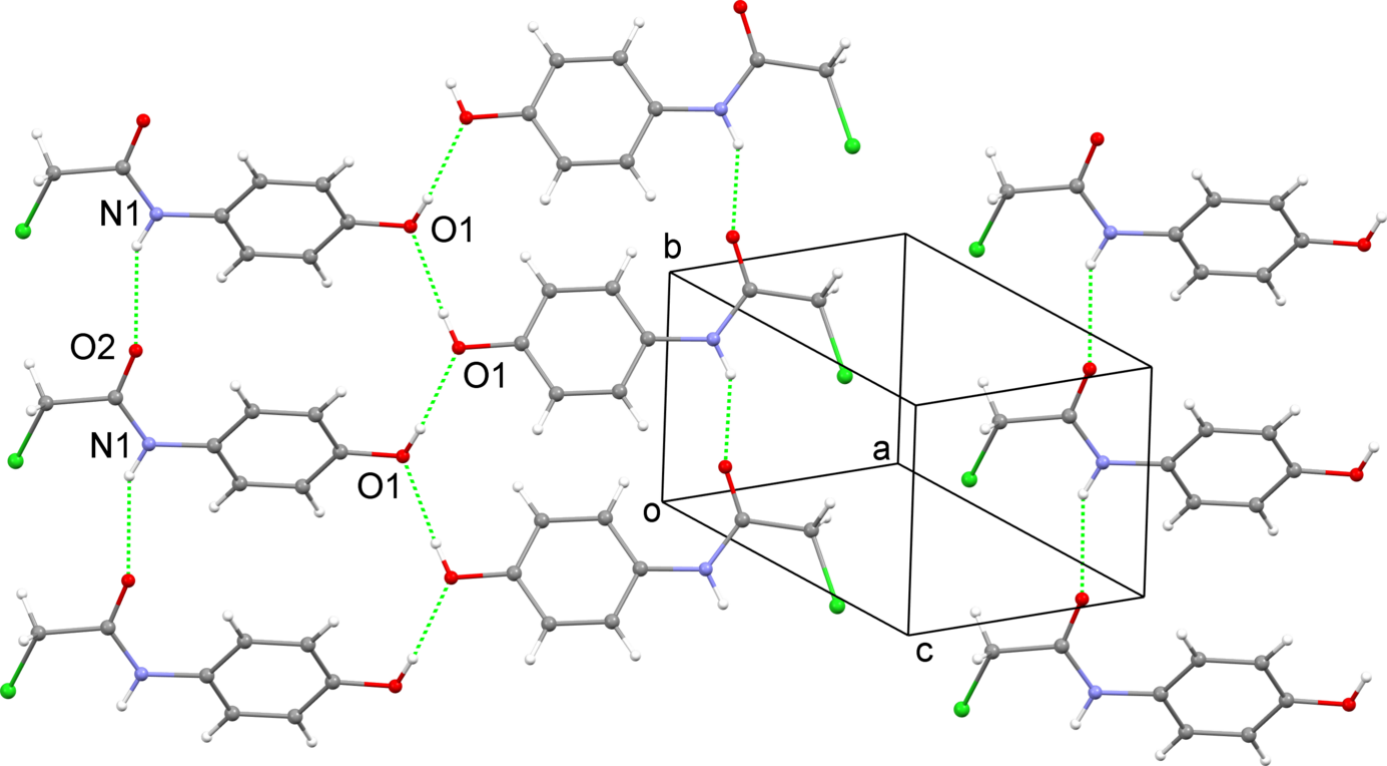
A segment of the packing in the crystal of 2-chloro-*N*-(4-hy­droxy­phen­yl)acetamide showing the inter­molecular N—H⋯O and O—H⋯O hydrogen bonds as green dotted lines.

In the crystal, neighbouring mol­ecules are linked by N—H⋯O hydrogen-bonding between translationally related acetamide groups with a N1—H1⋯O2^i^ [symmetry code: (i) *x*, *y* − 1, *z*] angle of 145° and a N1⋯O2^i^ distance of 3.025 (3) Å, Table 1 ▸, to form linear chains parallel to the *b* axis (Fig. 2 ▸). The mol­ecules are also bridged by O—H⋯O contacts with a O1—H1*A*⋯O1^ii^ [symmetry code: (ii) −*x* + 2, *y* + 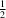, −*z* + 2] angle of 166° and an O1⋯O1^ii^ distance of 2.8585 (17) Å which, by themselves assemble mol­ecules along the 2_1_ screw axis in the *b*-axis direction. The combined hydrogen-bonding inter­actions result in almost flat tapes of mol­ecules parallel to [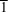
03].

## Synthesis and crystallization

4-Amino­phenol (1 mmol) was dissolved in pure acetic acid (30 ml) and placed in an ice-bath. Subsequently, chloro­acetyl chloride (1.2 mmol) was added portion-wise under stirring. At the end of the reaction, a solution of sodium acetate (25 ml) was added, and a solid precipitate formed after 30 min of stirring at room temperature. The resulting solid was filtered, washed with cold water, dried and recrystallized from its ethanol solution to yield the title compound as colourless crystals.

Yield = 89%, colour:colourless, m.p. = 413–415 K. FT–IR (ATR, ν, cm^−1^): 3385 (OH), 3200 (NH), 1640 (C=O). ^1^H NMR (500 MHz, DMSO-d_6_): δ p.p.m. 4.21 (*s*, 2H, CH_2_), 6.76–7.34 (*m*, 4H, Ar—H), 9.20 (*s*, 1H, OH), 10.23 (*s*, 1H, NH). ^13^C NMR (500 MHz, DMSO-*d*_6_): 43.42 (**C**H_2_); 117.68, 122.20, 131.50, 132.63, 153.68 (C—Ar); 164.76 (**C**=O). HRMS (ESI): calculated for C_8_H_8_ClNO_2_ [*M* - H]^+^ 186.0224, found 186.0328.

## Refinement

Crystal data, data collection and structure refinement details are summarized in Table 2 ▸.

## Supplementary Material

Crystal structure: contains datablock(s) I. DOI: 10.1107/S2414314624010150/tk4111sup1.cif

Structure factors: contains datablock(s) I. DOI: 10.1107/S2414314624010150/tk4111Isup2.hkl

Supporting information file. DOI: 10.1107/S2414314624010150/tk4111Isup3.cml

CCDC reference: 2392239

Additional supporting information:  crystallographic information; 3D view; checkCIF report

## Figures and Tables

**Table 1:** Hydrogen-bond geometry (Å, °)

*D*—H⋯*A*	*D*—H	H⋯*A*	*D*⋯*A*	*D*—H⋯*A*
C3—H3⋯O2	0.93	2.34	2.873 (4)	116
N1—H1⋯Cl1	0.86	2.53	2.999 (2)	115
N1—H1⋯O2^i^	0.86	2.28	3.025 (3)	145
O1—H1*A*⋯O1^ii^	0.82	2.06	2.8585 (17)	166

Symmetry codes: (i) 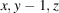; (ii) 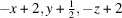.

**Table 2:** Experimental details

Crystal data	
Chemical formula	C_8_H_8_ClNO_2_
*M*_r_	185.60
Crystal system, space group	Monoclinic, *P*2_1_
Temperature (K)	296
*a*, *b*, *c* (Å)	6.5088 (6), 5.1758 (5), 12.2175 (14)
β (°)	101.649 (10)
*V* (Å^3^)	403.11 (7)
*Z*	2
Radiation type	Mo *K*α
μ (mm^−1^)	0.43
Crystal size (mm)	0.45 × 0.20 × 0.07

Data collection	
Diffractometer	SuperNova, Dual, Cu at home/near, Atlas
Absorption correction	Gaussian (*CrysAlis PRO*; Rigaku OD, 2023 ▸)
*T*_min_, *T*_max_	0.642, 1.000
No. of measured, independent and observed [*I* > 2σ(*I*)] reflections	3642, 1902, 1487
*R*_int_	0.028
(sin θ/λ)_max_ (Å^−1^)	0.697

Refinement	
*R*[*F*^2^ > 2σ(*F*^2^)], *wR*(*F*^2^), *S*	0.039, 0.081, 1.06
No. of reflections	1902
No. of parameters	110
No. of restraints	1
H-atom treatment	H-atom parameters constrained
Δρ_max_, Δρ_min_ (e Å^−3^)	0.16, −0.19
Absolute structure	Flack *x* determined using 510 quotients [(*I*^+^)−(*I*^−^)]/[(*I*^+^)+(*I*^−^)] (Parsons *et al.*, 2013 ▸)
Absolute structure parameter	0.11 (5)

Computer programs: *CrysAlis PRO* (Rigaku OD, 2023 ▸), *SHELXT* (Sheldrick, 2015*a* ▸), *SHELXL2019/2* (Sheldrick, 2015*b* ▸), *Mercury* (Macrae *et al.*, 2020 ▸) and *WinGX* (Farrugia, 2012 ▸).

## full crystallographic data

### 2-Chloro-N-(4-hydroxyphenyl)acetamide Crystal data

**Table:**

C_8_H_8_ClNO_2_	*F*(000) = 192
*M**_r_* = 185.60	*D*_x_ = 1.529 Mg m^−^^3^
Monoclinic, *P*2_1_	Mo *K*α radiation, λ = 0.71073 Å
*a* = 6.5088 (6) Å	Cell parameters from 1576 reflections
*b* = 5.1758 (5) Å	θ = 3.9–28.2°
*c* = 12.2175 (14) Å	µ = 0.43 mm^−^^1^
β = 101.649 (10)°	*T* = 296 K
*V* = 403.11 (7) Å^3^	Plate, colourless
*Z* = 2	0.45 × 0.20 × 0.07 mm

### 2-Chloro-N-(4-hydroxyphenyl)acetamide Data collection

**Table:**

SuperNova, Dual, Cu at home/near, Atlas diffractometer	1487 reflections with *I* > 2σ(*I*)
ω scans	*R*_int_ = 0.028
Absorption correction: gaussian (CrysAlis Pro; Rigaku OD, 2023)	θ_max_ = 29.7°, θ_min_ = 3.3°
*T*_min_ = 0.642, *T*_max_ = 1.000	*h* = −8→8
3642 measured reflections	*k* = −7→7
1902 independent reflections	*l* = −16→13

### 2-Chloro-N-(4-hydroxyphenyl)acetamide Refinement

**Table:**

Refinement on *F*^2^	Hydrogen site location: inferred from neighbouring sites
Least-squares matrix: full	H-atom parameters constrained
*R*[*F*^2^ > 2σ(*F*^2^)] = 0.039	*w* = 1/[σ^2^(*F*_o_^2^) + (0.0294*P*)^2^ + 0.0021*P*] where *P* = (*F*_o_^2^ + 2*F*_c_^2^)/3
*wR*(*F*^2^) = 0.081	(Δ/σ)_max_ < 0.001
*S* = 1.06	Δρ_max_ = 0.16 e Å^−^^3^
1902 reflections	Δρ_min_ = −0.18 e Å^−^^3^
110 parameters	Absolute structure: Flack *x* determined using 510 quotients [(*I*^+^)-(*I*^-^)]/[(*I*^+^)+(*I*^-^)] (Parsons *et al.*, 2013)
1 restraint	Absolute structure parameter: 0.11 (5)

### 2-Chloro-N-(4-hydroxyphenyl)acetamide Special details

**Table:**

Geometry. All esds (except the esd in the dihedral angle between two l.s. planes) are estimated using the full covariance matrix. The cell esds are taken into account individually in the estimation of esds in distances, angles and torsion angles; correlations between esds in cell parameters are only used when they are defined by crystal symmetry. An approximate (isotropic) treatment of cell esds is used for estimating esds involving l.s. planes.

### 2-Chloro-N-(4-hydroxyphenyl)acetamide Fractional atomic coordinates and isotropic or equivalent isotropic displacement parameters (Å^2^)

**Table:**

	*x*	*y*	*z*	*U*_iso_*/*U*_eq_	
C1	0.7301 (4)	0.3699 (6)	0.8980 (2)	0.0311 (7)	
C2	0.5982 (4)	0.5654 (6)	0.9179 (3)	0.0334 (7)	
H2	0.645995	0.688449	0.972562	0.040*	
C3	0.3940 (5)	0.5786 (6)	0.8563 (3)	0.0337 (7)	
H3	0.305127	0.710757	0.869335	0.040*	
C4	0.3236 (4)	0.3932 (6)	0.7754 (2)	0.0290 (7)	
C5	0.4562 (5)	0.1964 (6)	0.7575 (3)	0.0335 (8)	
H5	0.408280	0.070786	0.703994	0.040*	
C6	0.6593 (5)	0.1844 (6)	0.8184 (3)	0.0348 (8)	
H6	0.747984	0.051642	0.805778	0.042*	
C7	−0.0065 (5)	0.6041 (7)	0.6835 (3)	0.0349 (7)	
C8	−0.2181 (5)	0.5695 (7)	0.6051 (3)	0.0442 (9)	
H8A	−0.238963	0.713348	0.553173	0.053*	
H8B	−0.326264	0.580067	0.649112	0.053*	
N1	0.1164 (3)	0.3959 (5)	0.7083 (2)	0.0331 (6)	
H1	0.065971	0.250333	0.681401	0.040*	
O1	0.9342 (3)	0.3507 (4)	0.9570 (2)	0.0433 (6)	
H1A	0.967245	0.484830	0.991810	0.065*	
O2	0.0363 (3)	0.8215 (4)	0.7186 (2)	0.0500 (6)	
Cl1	−0.25580 (12)	0.27889 (19)	0.52655 (7)	0.0555 (3)	

### 2-Chloro-N-(4-hydroxyphenyl)acetamide Atomic displacement parameters (Å^2^)

**Table:**

	*U*^11^	*U*^22^	*U*^33^	*U*^12^	*U*^13^	*U*^23^
C1	0.0250 (14)	0.0296 (17)	0.0360 (17)	−0.0017 (13)	−0.0002 (12)	0.0054 (14)
C2	0.0334 (17)	0.0289 (17)	0.0344 (17)	−0.0011 (14)	−0.0014 (13)	−0.0042 (14)
C3	0.0304 (16)	0.0303 (17)	0.0383 (18)	0.0034 (14)	0.0022 (13)	−0.0025 (14)
C4	0.0251 (15)	0.0268 (15)	0.0329 (16)	−0.0010 (13)	0.0006 (12)	0.0036 (13)
C5	0.0326 (16)	0.0266 (17)	0.0386 (18)	−0.0022 (13)	0.0007 (13)	−0.0037 (13)
C6	0.0293 (15)	0.0254 (16)	0.050 (2)	0.0063 (12)	0.0079 (14)	−0.0008 (14)
C7	0.0279 (16)	0.0337 (18)	0.0412 (18)	0.0000 (14)	0.0022 (13)	0.0041 (15)
C8	0.0340 (17)	0.0336 (19)	0.058 (2)	0.0014 (15)	−0.0066 (15)	0.0046 (17)
N1	0.0279 (13)	0.0259 (13)	0.0407 (15)	−0.0005 (11)	−0.0047 (11)	−0.0030 (12)
O1	0.0293 (10)	0.0387 (15)	0.0545 (14)	0.0005 (10)	−0.0093 (9)	−0.0015 (12)
O2	0.0407 (12)	0.0297 (14)	0.0705 (16)	0.0019 (11)	−0.0105 (11)	−0.0033 (12)
Cl1	0.0505 (5)	0.0519 (5)	0.0530 (5)	−0.0009 (5)	−0.0155 (4)	−0.0056 (5)

### 2-Chloro-N-(4-hydroxyphenyl)acetamide Geometric parameters (Å, º)

**Table:**

C1—C6	1.378 (4)	C5—H5	0.9300
C1—C2	1.379 (4)	C6—H6	0.9300
C1—O1	1.381 (3)	C7—O2	1.216 (4)
C2—C3	1.391 (4)	C7—N1	1.340 (4)
C2—H2	0.9300	C7—C8	1.520 (4)
C3—C4	1.387 (4)	C8—Cl1	1.774 (4)
C3—H3	0.9300	C8—H8A	0.9700
C4—C5	1.381 (4)	C8—H8B	0.9700
C4—N1	1.430 (3)	N1—H1	0.8600
C5—C6	1.382 (4)	O1—H1A	0.8200
			
C6—C1—C2	120.3 (3)	C1—C6—H6	120.1
C6—C1—O1	117.8 (3)	C5—C6—H6	120.1
C2—C1—O1	121.9 (3)	O2—C7—N1	125.5 (3)
C1—C2—C3	120.1 (3)	O2—C7—C8	116.4 (3)
C1—C2—H2	119.9	N1—C7—C8	118.1 (3)
C3—C2—H2	119.9	C7—C8—Cl1	116.7 (2)
C4—C3—C2	119.6 (3)	C7—C8—H8A	108.1
C4—C3—H3	120.2	Cl1—C8—H8A	108.1
C2—C3—H3	120.2	C7—C8—H8B	108.1
C5—C4—C3	119.8 (3)	Cl1—C8—H8B	108.1
C5—C4—N1	117.6 (3)	H8A—C8—H8B	107.3
C3—C4—N1	122.6 (3)	C7—N1—C4	126.0 (3)
C4—C5—C6	120.6 (3)	C7—N1—H1	117.0
C4—C5—H5	119.7	C4—N1—H1	117.0
C6—C5—H5	119.7	C1—O1—H1A	109.5
C1—C6—C5	119.7 (3)		
			
N1—C7—C8—Cl1	−15.4 (4)		

### 2-Chloro-N-(4-hydroxyphenyl)acetamide Hydrogen-bond geometry (Å, º)

**Table:**

*D*—H···*A*	*D*—H	H···*A*	*D*···*A*	*D*—H···*A*
C3—H3···O2	0.93	2.34	2.873 (4)	116
N1—H1···Cl1	0.86	2.53	2.999 (2)	115
N1—H1···O2^i^	0.86	2.28	3.025 (3)	145
O1—H1*A*···O1^ii^	0.82	2.06	2.8585 (17)	166

Symmetry codes: (i) *x*, *y*−1, *z*; (ii) −*x*+2, *y*+1/2, −*z*+2.
